# Differences in the intrahepatic expression of immune checkpoint molecules on T cells and natural killer cells in chronic HBV patients

**DOI:** 10.3389/fimmu.2024.1489770

**Published:** 2025-01-15

**Authors:** Lucile Dumolard, Marie-Noelle Hilleret, Charlotte Costentin, Marion Mercey-Ressejac, Nathalie Sturm, Theophile Gerster, Thomas Decaens, Evelyne Jouvin-Marche, Patrice N. Marche, Zuzana Macek Jilkova

**Affiliations:** ^1^ Univ. Grenoble Alpes, Inserm U 1209, CNRS UMR 5309, Institute for Advanced Biosciences, Grenoble, France; ^2^ Service d’hépato-gastroentérologie, Pôle Digidune, CHU Grenoble Alpes, La Tronche, France; ^3^ Service d’anatomie et de cytologie pathologiques, CHU Grenoble Alpes, Grenoble, France; ^4^ Translational Research in Autoimmunity and Inflammation Group (TRAIG), Translational Innovation in Medicine and Complexity (TIMC), University Grenoble-Alpes, CNRS Unité mixte de recherche (UMR) 5525, La Tronche, France

**Keywords:** HBV, PD-1, 4-1BB, immune checkpoint molecules, liver, T cells, NK cells

## Abstract

**Background:**

Patients with chronic hepatitis B virus (HBV) infection are characterized by impaired immune response that fails to eliminate HBV. Immune checkpoint molecules (ICMs) control the amplitude of the activation and function of immune cells, which makes them the key regulators of immune response.

**Methods:**

We performed a multiparametric flow cytometry analysis of ICMs and determined their expression on intrahepatic lymphocyte subsets in untreated and treated patients with HBV in comparison with non-pathological liver tissue.

**Results:**

The liver of untreated HBV patients exhibited a high accumulation of PD-1^+^CD8^+^ T cells, while the frequencies of 4-1BB^+^ T cells, 4-1BB^+^ natural killer (NK) cells, and TIM-3^+^CD8^+^ T cells were the highest in the chronic hepatitis phase. Our findings showed that the HBeAg status is linked to a distinct immune phenotype of intrahepatic CD8^+^ T cells and NK cells characterized by high expression of ICMs, particularly 4-1BB. Importantly, antiviral treatment partially restored the normal expression of ICMs. Finally, we described important differences in ICM expression between intrahepatic and circulating NK cells in HBV patients.

**Conclusions:**

Our study shows clear differences in the intrahepatic expression of ICMs on NK cells and T cells in chronic HBV patients depending on their clinical stage.

## 1 Introduction

Hepatitis B is one of the most frequent forms of viral hepatitis globally and a significant cause of liver-related mortality. It has been estimated that in 2019, 295.9 million people worldwide were chronically infected with hepatitis B virus (HBV), leading to an estimated 821,100 deaths from liver cirrhosis, liver failure, and hepatocellular carcinoma (1).

Patients with chronic HBV (cHBV) are unable to develop an immune response that would be sufficiently robust, functional, and sustained to eliminate the infection (2, 3). Nucleotide or nucleoside analogs (NUCs) are currently the frequently applied therapeutics for cHBV. Antiviral agents like tenofovir and entecavir effectively inhibit the replication of HBV in almost all chronic patients, but hepatitis B surface antigen (HBsAg) loss, which reflects the functional clearance of the virus, is rarely achieved (4). Therefore, long-term or even life-long NUC therapy is required to maintain HBV suppression (4, 5).

Immunological control of HBV infection is critical for the functioning and permanent elimination of the virus. Thus, restoring adequate immune responses to the virus represents a major challenge that can lead to a promising therapeutic strategy (6). CD4^+^ T cells, CD8^+^ T cells, and natural killer (NK) cells play critical roles in the immune response to HBV infection (7). CD4^+^ T cells are essential for orchestrating the immune response, helping activate CD8^+^ T cells, and providing help for B-cell antibody production. Intrahepatic CD8^+^ T cells, mainly the population of HBV-specific CD8^+^ T cells, play a major role in the resolution of HBV chronic infection via their cytolytic and non-cytolytic effector functions. Similarly, NK cells, which are enriched in the liver, are crucial for antiviral immune response, as they can identify and directly kill HBV virus-infected cells with the release of cytotoxic granules and antiviral cytokines, without major histocompatibility complex restriction (8). However, in cHBV infection, both T cells and NK cells have shown an impairment in their function and activity (7, 9, 10).

The antiviral capacity of both T cells and NK cells depends on complex crosstalk within the liver microenvironment, and the expression of immune checkpoint molecules (ICMs) plays a critical role. The upregulation of inhibitory ICMs, such are programmed cell death 1 (PD-1) or T-cell immunoglobulin domain and mucin domain-3 (Tim-3) receptor, contributes to the exhaustion of effector cells that become unable to effectively eliminate the virus and thus favors the chronicity of the disease (11) but may also be important in avoiding excessive immune activation and thereby limiting immune-mediated liver damage. However, stimulatory ICMs, such as 4-1BB and OX40, offer potential avenues to enhance immune responses by restoring T-cell functionality and promoting antiviral activity. Overall, the interplay between inhibitory and stimulatory checkpoint molecules in cHBV infection plays a crucial role in shaping immune responses (10).

Several studies demonstrated that the expression of the inhibitory receptor PD-1 is significantly upregulated on HBV-specific T cells in cHBV infection (12–14), highlighting the role of immune exhaustion in the dysfunction of these cells. In fact, PD-1 expression is associated with diminished proliferative capacity, cytokine production, and cytotoxic activity of HBV-specific CD8^+^ T cells (12, 13). This immune dysfunction is one of the key factors in the pathogenesis of cHBV infection, allowing the virus to evade immune clearance and sustain long-term infection. Blocking the PD-1/PD-L1 axis could restore the reactivity of T cells, mainly the capacity of HBV-specific T-cell populations to expand and to produce cytokines, which could lead to the elimination of HBV (12, 13, 15–17).

The role of PD-1 expression on intrahepatic and circulating T cells in the context of HBV infection is well recognized, and several studies have also explored the modulation of other ICMs on lymphocytes of cHBV-infected patients (10). While much of this research has focused on circulating lymphocytes, there is less information about ICM expression in the intrahepatic environment, despite its importance. Intrahepatic immune cells are unique, with CD8^+^ T cells and NK cells being more prevalent in the liver than in circulation. Their distinct capacity to express specific ICMs could significantly influence antiviral responses. Recent advances, including single-cell RNA sequencing, have shed light on ICM expression in intrahepatic T cells, offering valuable insights into liver-specific immune regulation during HBV infection (18, 19). Although this costly technique can only be currently used on a very limited number of samples, the results of Zhang et al. indicate that each phase of HBV infection may be associated with a unique intrahepatic immune signature (19). In addition, a recent study by Andreata et al. underscores the importance of stimulatory ICMs in cHBV infection. Their research using HBV-transgenic mice demonstrated that 4-1BB agonism is a promising strategy for converting dysfunctional effector cells into antiviral effectors for treating cHBV infections (20).

For developing new therapies to eradicate HBV, it is essential to describe the expression of ICMs during different phases of HBV in patients, as well as whether and how the NUC treatment modulates the frequency, activity, and ICM expression of intrahepatic NK and T cells. HBV infection is a dynamic disease that reflects the ongoing interaction between HBV and the host’s immune system over time. Patients with cHBV infection can be classified based on viral activity and liver disease markers. HBV patients in the chronic infection phase usually exhibit normal alanine aminotransferase (ALT) levels, minimal or no liver inflammation, and low fibrosis. In contrast, the chronic hepatitis phase is marked by intermediate to high levels of HBsAg, elevated ALT, and moderate-to-severe liver disease (21). In this study, we present a multiparametric analysis of immune checkpoint expression on intrahepatic and circulating lymphocyte subsets in both untreated HBV patients across different phases of infection and treated HBV-infected patients. By comparing these findings with normal liver tissue, we highlight the link between intrahepatic immune checkpoint expression and the clinical stage of the patients. Our findings provide novel insights into the immune landscape of intrahepatic T and NK cells in the setting of chronic HBV infection, offering contributions to the understanding of immune regulation in this disease.

## 2 Materials and methods

### 2.1 Patients and liver biopsy processing

This study includes 40 patients who were enrolled between 2017 and 2021 either at diagnosis prior to any treatment initiation or during clinical follow-up (Department of Gastroenterology and Hepatology, CHU Grenoble-Alpes). All patients with HCV or HIV infection, as well as those with alcohol abuse/dependency, were excluded. Detailed patient characteristics have been recorded as shown in **Table 1**. The HBV cohort consists of groups of untreated HBV patients: chronic infection (n = 9), chronic hepatitis (n = 16), and NUC-treated HBV patients (n = 5). Patients were divided by hepatologists (MN.H., C.C., and T.D.) based on HBV markers (HBsAg, HBeAg/anti-HBe, and HBV DNA) and liver disease parameters [ALT, aspartate aminotransferase (AST), and fibrosis] as recommended (21). Untreated HBV patients had undergone core needle liver biopsy as part of the diagnosis. NUC-treated HBV patients had undergone liver biopsy as part of medical follow-up to justify possible NUC treatment withdrawal. The mean time between the start of treatment and liver biopsy in the group of NUC-treated HBV patients was 3.1 years. The control group includes core needle liver biopsies from patients with suspected non-alcoholic fatty liver disease (NAFLD) where NAFLD was not confirmed (liver steatosis <5%) (n = 10). Controls had normal ALT and AST levels and no liver inflammation or fibrosis (**Table 1**).

**Table 1:** Clinical, biological, and histological features of patients.

Parameters	Controls n = 10	Chronic infection (former inactive carriers) n = 9	Chronic hepatitis n = 16	NUC-treated* n = 5
Sex (M/F)	5/5	5/4	14/2	3/2
Age, median [IQR]	43.0 [36.0–57.5]	35.0 [19.8–48.3]	29.0 [19.3–30.8]	58.0 [42.0–70.5]
ALT (U/L), median [IQR]	33.0 [26.8–40.8]	32.0 [21.5–40.0]	53.0 [41.3–65.5]	49.5 [29.5–56.0]
AST (U/L), median [IQR]	27.0 [23.8–32.3]	29.0 [24.5–33.0]	41.0 [31.0–48.3]	32.5 [14.5–48.3]
HBeAg, n (%) Negative Positive	10 (100%) 0 (0%)	9 (100%) 0 (0%)	11 (69%) 5 (31%)	5 (100%) 0 (0%)
HBsAg (UI/mL), median [IQR]	0	3,363 [1,543–6,064]	14,846 [4,333–25,357]	396 [91–1,000]
HBV DNA (log_10_ UI/mL), median [IQR]	NA	3.13 [1.94–3.55]	4.1 [3.33–6.52]	1.00 [1.00–1.89]
Antiviral treatment Entecavir Tenofovir	0 0	0 0	0 0	2 3
Stage of fibrosis F0/F1/F2/F3/F4	10/0/0/0/0	1/8/0/0/0	3/8/4/1/0	0/2/0/1/2
Activity score A0/A1/A2/A3	10/0/0/0	3/6/0/0	0/14/1/1	3/0/1/1

Values are presented as median [IQR, 25th–75th percentile].

ALT, alanine transaminase; AST, aspartate transaminase; HBeAg, hepatitis Be antigen; HBsAg, hepatitis B surface antigen; NA, not applicable; IQR, interquartile range; NUC, nucleotide or nucleoside analog.

*The time between start of treatment and liver biopsy in group of NUC-treated HBV patients was 3.1 years.

Liver biopsies were divided into two parts: one part was used for histological examination, assessed by experienced liver pathologists, whereas the other part was processed within 1 hour following the clinical biopsy to conduct extensive phenotypic immunological analyses. Paired peripheral blood samples and biopsies were obtained in 16 HBV patients (**Supplementary Table S1**). The study was performed in accordance with the Declaration of Helsinki and the French legislation based on local sample collection (DC-2014-2295) and approved by the Ethics Committee of CHU Grenoble-Alpes: patient collection number AC-2019-3627 (CRB03) and the biological resource center of CHU Grenoble-Alpes (nBRIF BB-0033-00069). All participants provided written informed consent.

### 2.2 Flow cytometry analyses

Immediately after the needle liver biopsy, freshly harvested liver tissue was transferred in the HypoThermosol™ FRS solution (StemCell, Vancouver, BC, Canada; 4°C) and weighed, and cells were recovered through mechanical disruption as previously described (22, 23). Fresh intrahepatic cell suspension and blood samples were divided into two tubes and stained, without any stimulation, with the anti-human antibodies (see **Supplementary Information**: Materials and methods). The gating strategy and fluorescence minus one (FMO) controls (**Supplementary Figures S1**, **S2**) allowed us to separate the main populations of lymphocytes and determine positive cells.

### 2.3 Statistical analysis

Analyses were performed using the statistical software GraphPad Prism 9 (GraphPad Software, La Jolla, CA, USA). Normal distribution was tested by means of the D’Agostino–Pearson omnibus normality test. The non-parametric Kruskal–Wallis one-way analysis of variance was used for multiple comparisons of non-parametric data. p-Values in the correlation matrix were adjusted using the Bonferroni correction method. Spearman’s correlation non-parametric test was conducted to determine the degree of correlation between variables. For non-parametric paired data, the Wilcoxon matched-pairs signed-rank test was used. The Mann–Whitney test was used to compare unpaired data of two groups. p-Value <0.05 was considered to be significant.

## 3 Results

### 3.1 The number and distribution of intrahepatic lymphocytes are modified in HBV

**Figure 1:**
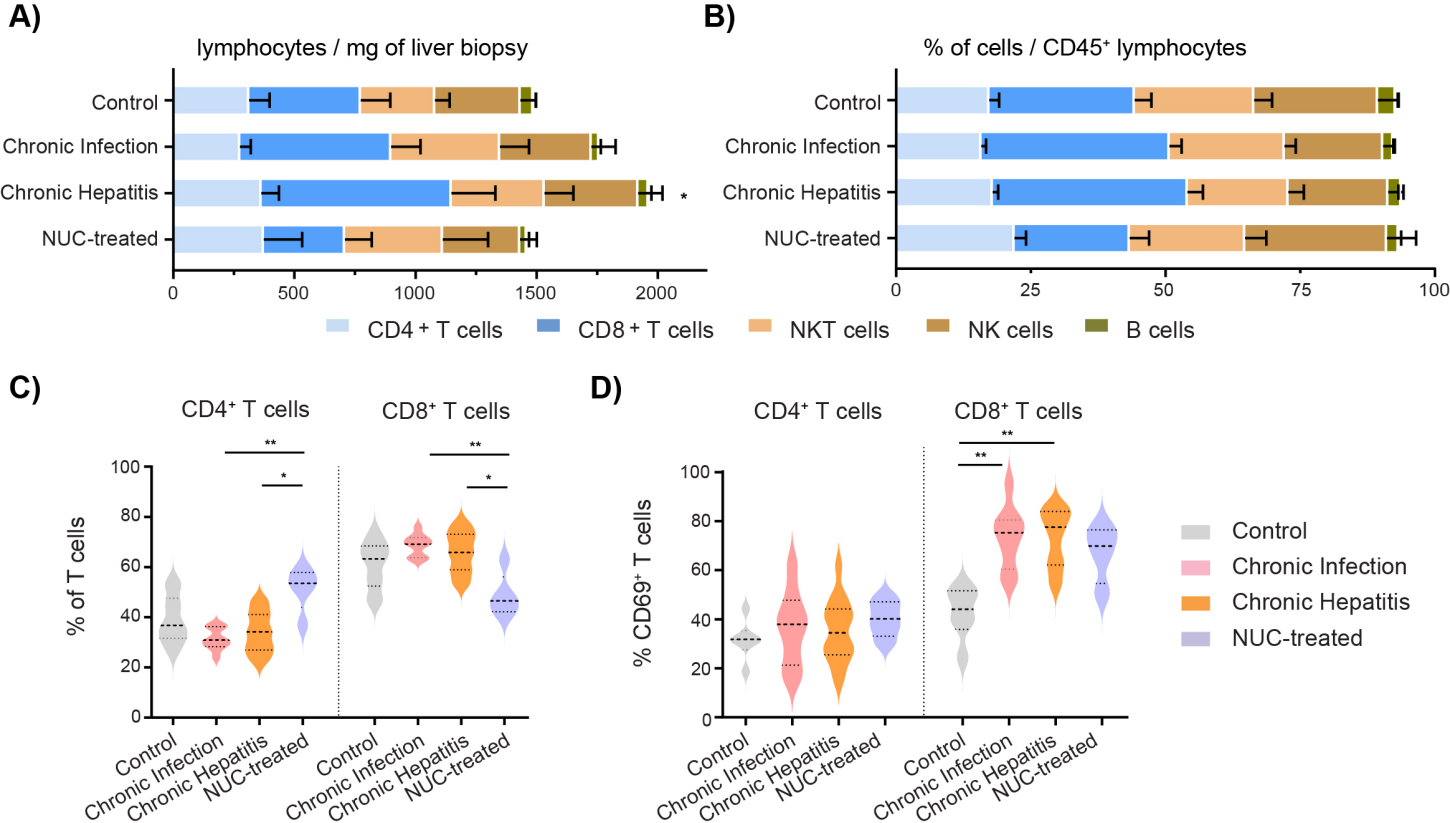
Frequency and the number of intrahepatic immune cells in HBV patients. **(A)** Number of intrahepatic immune cells per mg of liver tissue. *p < 0.05 compared to control, Kruskal–Wallis test with Dunn’s multiple comparison post-test. **(B)** Distribution of intrahepatic immune cells in CD45^+^ lymphocyte population. Data are expressed as mean ± SEM. **(C)** Frequency of intrahepatic CD4^+^ T cells and CD8^+^ T cells in T-cell population. **(D)** Frequency of intrahepatic CD69^+^ cells in T-cell population. *p < 0.05, **p < 0.01, between groups, Kruskal–Wallis test with Dunn’s multiple comparison post-test. Data are visualized as violin plots showing median and quartile data. Control (n = 10), untreated chronic infection (n = 9) and chronic hepatitis (n = 16), and NUC-treated HBV (n = 5). HBV, hepatitis B virus; NUC, nucleotide or nucleoside analog.

We analyzed by multiparametric flow cytometry fresh liver biopsies and divided them into groups: control (n = 10), untreated cHBV patients characterized as chronic infection (n = 9) or chronic hepatitis (n = 16), and NUC-treated HBV (n = 5). We focused on T, NK, NKT, and B cells, adopting the strategy with the principle of gating (**Supplementary Figure S1**), as previously described (24, 25). We calculated the estimated number of cells per mg of tissue based on the original weight of the liver biopsy, and we determined the frequency of immune cell subsets as a frequency per CD45^+^ lymphocytes (**Supplementary Table S2**). The numbers of cells per mg reflect total cell infiltration, while frequencies capture immune profile alterations within the specific intrahepatic immune cell population. As expected, the number of lymphocytes per mg of tissue was increased in untreated chronic hepatitis patients compared to the control group (mean ± SE: 1,961.7 ± 421.5 vs. 1,486.2 ± 307.9, p = 0.0482). This was mainly caused by the accumulation of CD8^+^ T cells in untreated chronic hepatitis (832.6 ± 185.3) compared to control (460.1 ± 122.4, p = 0.0486) and NUC-treated HBV patients (334.4 ± 111.9, p = 0.2199) (**Figure 1A**). The overall distribution of intrahepatic immune cells per CD45^+^ population showed a tendency of higher frequency of CD8^+^ T cells in chronic infection (34.9% ± 2.2%) and chronic hepatitis (36.2% ± 2.8%) compared to control liver (27.0% ± 3.1%) and the NUC-treated HBV group (21.40% ± 3.6%) (**Figure 1B**). When the frequency of CD4^+^ and CD8^+^ cells was quantified per T-cell population, we observed a significant increase of CD4^+^ and a decrease of CD8^+^ T cells in the NUC-treated HBV group compared to untreated HBV patients (**Figure 1C**). Importantly, the higher frequency of CD8^+^ T cells in chronic infection and chronic hepatitis patients was associated with a significant increase of activation marker CD69 [median 75.3%, interquartile range (IQR) 60.4–80.5; median 77.5%, IQR 62.2–83.9] compared to control (44.1%, IQR 35.8–51.6; p = 0.0093 and p = 0.0020) (**Figure 1D**).

### 3.2 Expression of ICMs on intrahepatic lymphocytes is modulated in HBV patients

**Figure 2:**
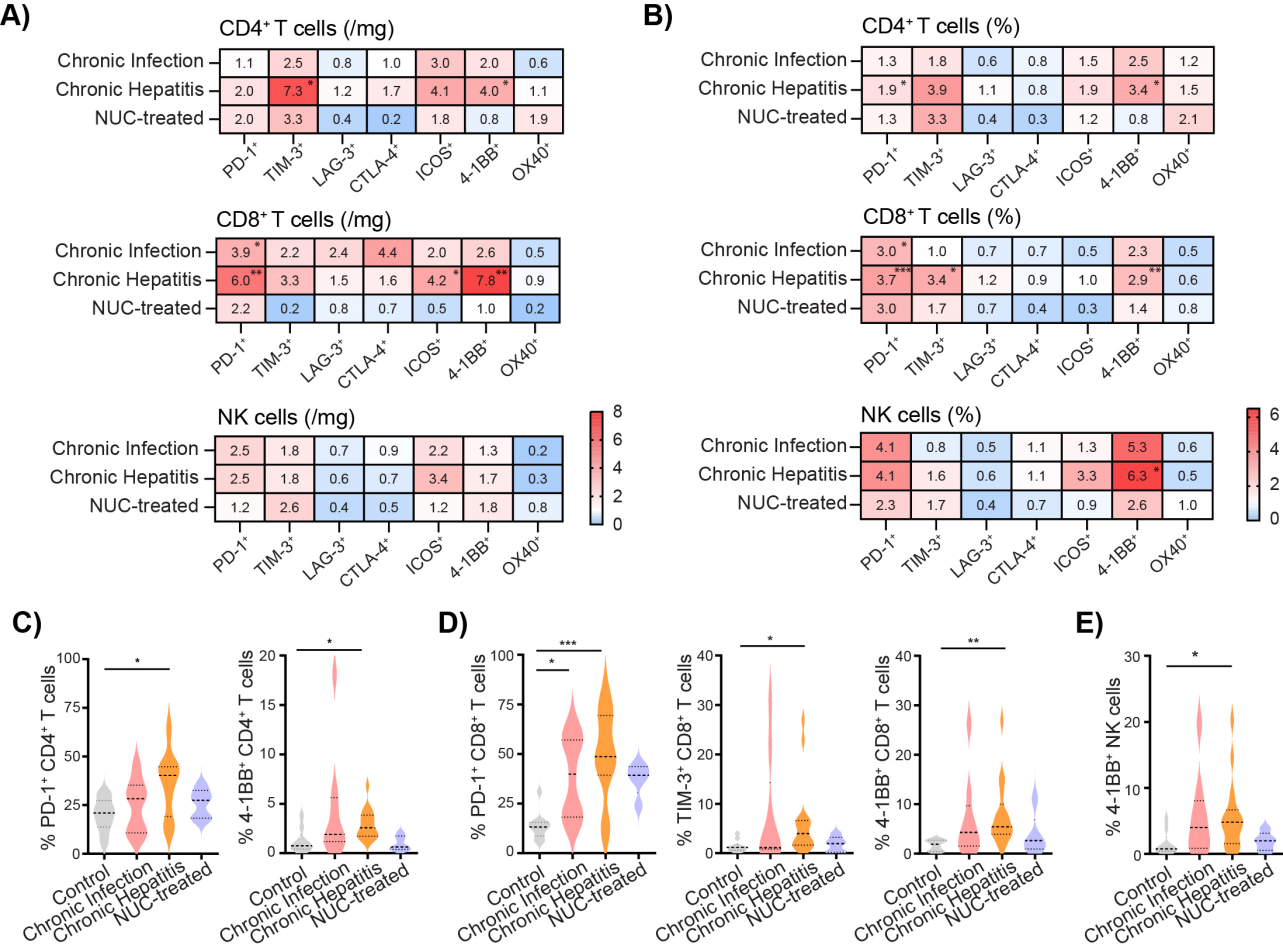
Expression of ICMs on intrahepatic lymphocytes is modulated in HBV patients. **(A)** Heatmap of fold change of number of intrahepatic immune cells expressing ICMs per mg of tissue. Control group set as 1. **(B)** Heatmap of fold change of median of frequency of intrahepatic immune cells expressing ICMs. Control set as 1. **(C)** The frequency of 4-1BB^+^ cells per CD4^+^ T cells. **(D)** The frequency of PD-1^+^, TIM-3^+^ cells, and 4-1BB^+^ cells per CD8^+^ T cells. **(E)** The frequency of 4-1BB^+^ NK cells. Data are visualized as violin plots showing median and quartile data. Control (n = 10), untreated chronic infection (n = 9) and chronic hepatitis (n = 16), and NUC-treated HBV (n = 5). *p < 0.05, **p < 0.01, ***p < 0.001 between groups (Kruskal–Wallis test with Dunn’s multiple comparison post-test). ICMs, immune checkpoint molecules; HBV, hepatitis B virus; NUC, nucleotide or nucleoside analog.

Next, we analyzed the expression of ICMs on the cell surface of CD4^+^ T cells, CD8^+^ T cells, and NK cells and compared them between the control group (set as 1) and the HBV-infected patients. The heatmaps are based on the median of the fold change in the number (**Figure 2A**) and the frequency (**Figure 2B**) of intrahepatic lymphocytes expressing PD-1, TIM-3, LAG-3, CTLA-4, ICOS, 4-1BB, and OX40 molecules. Our results revealed an accumulation of intrahepatic CD4^+^ T cells expressing TIM-3 and 4-1BB in the chronic hepatitis group compared to the control liver (**Figure 2A**). The number of PD-1^+^CD8^+^ T cells was highly increased in chronic infection as well as in the chronic hepatitis group, while the ICOS and 4-1BB-positive CD8^+^ T cells were enriched only in the liver of chronic hepatitis patients compared to control.

Data expressed as the frequency of the cells per the lymphocyte population revealed a significantly increased frequency of PD-1^+^CD4^+^ T and 4-1BB^+^CD4^+^ T cells in the chronic hepatitis group compared to the control liver (**Figures 2B, C**). The frequency of PD-1^+^CD8^+^ T cells was highly increased in both the chronic infection and chronic hepatitis groups, whereas TIM-3^+^CD8^+^ T and 4-1BB^+^CD8^+^ T cells were significantly higher only in the liver of chronic hepatitis patients compared to control (**Figures 2B, D**). Interestingly, our results also revealed a tendency of increased expression of intrahepatic PD-1^+^ and ICOS^+^ NK cells (**Supplementary Figures S3A, B**) and mainly the significant increase of 4-1BB^+^ NK cells in the chronic hepatitis group compared to control (**Figures 2B, E**).

Thus, the liver environment of untreated chronic infection as well as chronic hepatitis patients is characterized by a high accumulation of CD8^+^ T cells expressing CD69 and PD-1. Furthermore, intrahepatic lymphocytes during the chronic hepatitis phase exhibit an increased expression of TIM-3 and 4-1BB.

### 3.3 HBeAg status correlates with intrahepatic immune profile of T and NK cells

**Figure 3:**
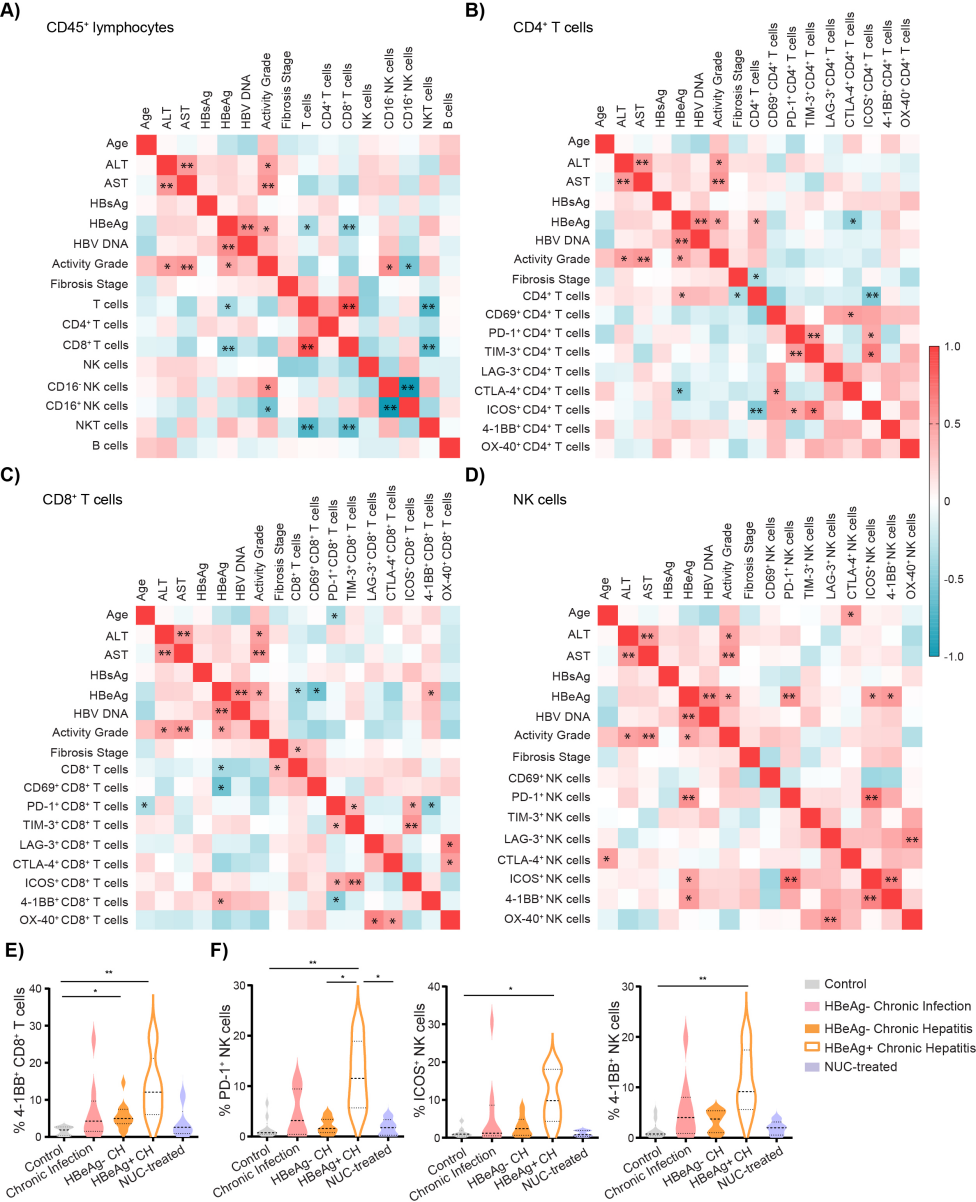
Intrahepatic lymphocyte characteristics correlate with cHBV patient clinical characteristics. **(A)** Heatmap of correlation of patient characteristics and frequencies of intrahepatic immune cells per CD45^+^ lymphocytes in untreated HBV patients. **(B)** Heatmap of correlation of patient characteristics and frequencies of intrahepatic CD4^+^ T cells expressing immune ICMs. **(C)** Heatmap of correlation of patient characteristics and frequencies of intrahepatic CD8^+^ T cells expressing ICMs. **(D)** Heatmap of correlation of patient characteristics and frequencies of intrahepatic NK cells expressing ICMs. Color corresponds to Spearman’s correlation coefficient r, n = 25. *p < 0.05 and **p < 0.01 represent statistically significant correlations. **(E)** The frequency of 4-1BB^+^ cells per CD8^+^ T cells. **(F)** The frequency of PD-1^+^, ICOS^+^, and 4-1BB^+^ NK cells. Data are visualized as violin plots showing median and quartile data. Control (n = 10), untreated chronic infection (n = 9), HBeAg^−^ chronic hepatitis (n = 11), HBeAg^+^ chronic hepatitis (n = 5), and NUC-treated HBV (n = 5). Data are visualized as the violin plots showing median and quartile data. Each circle represents a patient. *p < 0.05, **p < 0.01 between groups (Kruskal–Wallis test with Dunn’s multiple comparison post-test). cHBV, chronic hepatitis B virus; ICMs, immune checkpoint molecules.

Next, we investigated the possible association between clinical data and lymphocyte characteristics in the group of untreated HBV patients. On the whole, the main parameter that correlated with the clinical data of patients was the HBeAg status. First, the analysis revealed that HBeAg-positive status was negatively correlated with the frequency of CD8^+^ T cells per CD45^+^ lymphocytes: r = −0.57, p < 0.0001 (**Figure 3A**). In contrast, the frequency of CD4^+^ per T cells correlated positively with HBeAg status (**Figure 3B**). Furthermore, HBeAg-positive status was negatively correlated with the frequency of activated CD69^+^CD8^+^ T cells (**Figure 3C**) but positively associated with the frequencies of 4-1BB^+^CD8^+^ T cells (**Figure 3C**). Subsequently, we observed a positive association between HBeAg status and the frequencies of PD1^+^, ICOS^+,^ and 4-1BB^+^ NK cells (**Figure 3D**). The frequency of CD16^−^ NK cells was positively associated with inflammation activity grade (**Figure 3A**).

Given the importance of HBeAg status to the phenotype of intrahepatic T cells and NK cells, we performed the subgroup statistical analysis dividing chronic hepatitis patients into HBeAg^−^ and HBeAg^+^ (**Supplementary Table S3**). Phenotyping revealed that the intrahepatic CD8^+^ T-cell population in HBeAg^+^ chronic hepatitis patients is characterized by the highest frequency of 4-1BB^+^ cells (**Figure 3E**). Interestingly, HBeAg^+^ patients have also a higher frequency of intrahepatic PD-1^+^ NK cells compared to control, untreated HBeAg^−^ chronic hepatitis patients, or NUC-treated patients (**Figure 3F**). Similarly, the frequency of ICOS^+^ NK as well as 4-1BB^+^ NK cells was higher in untreated HBeAg^+^ patients compared to control (**Figure 3F**).

Altogether, our results highlighted that the patients’ HBeAg status is associated with the specific immune phenotype of intrahepatic CD8^+^ T cells and NK cells, with high expression of ICMs, mainly 4-1BB.

Furthermore, the analysis revealed the correlation between the frequency of PD-1^+^ and TIM-3^+^CD4^+^ T cells (r = 0.514, p = 0.007) and PD-1^+^ and TIM-3^+^CD8^+^ T cells (r = 0.427, p = 0.03), suggesting potential co-expression of these ICMs and an exhausted phenotype of these cell populations (**Figures 3B, C**). Notably, this phenomenon was specific to T cells, as we observed no significant correlation between the frequency of PD-1^+^ and TIM-3^+^ NK cells (**Figure 3D**). However, a significant correlation was evident between the frequency of PD-1^+^ and ICOS^+^ NK cells (r = 0.718, p < 0.001), 4-1BB^+^ and ICOS^+^ NK cells (r = 0.623, p = 0.001), and OX-40^+^ and LAG-3^+^ NK cells (r = 0.577, p = 0.002) (**Figure 3D**). Additionally, in both CD4 and CD8 T-cell populations, we observed correlations between the frequencies of ICOS^+^ cells and PD-1^+^ and TIM-3^+^ T cells (**Figures 3B, C**).

### 3.4 Association between intrahepatic and circulating ICM expression in chronic HBV patients

**Figure 4:**
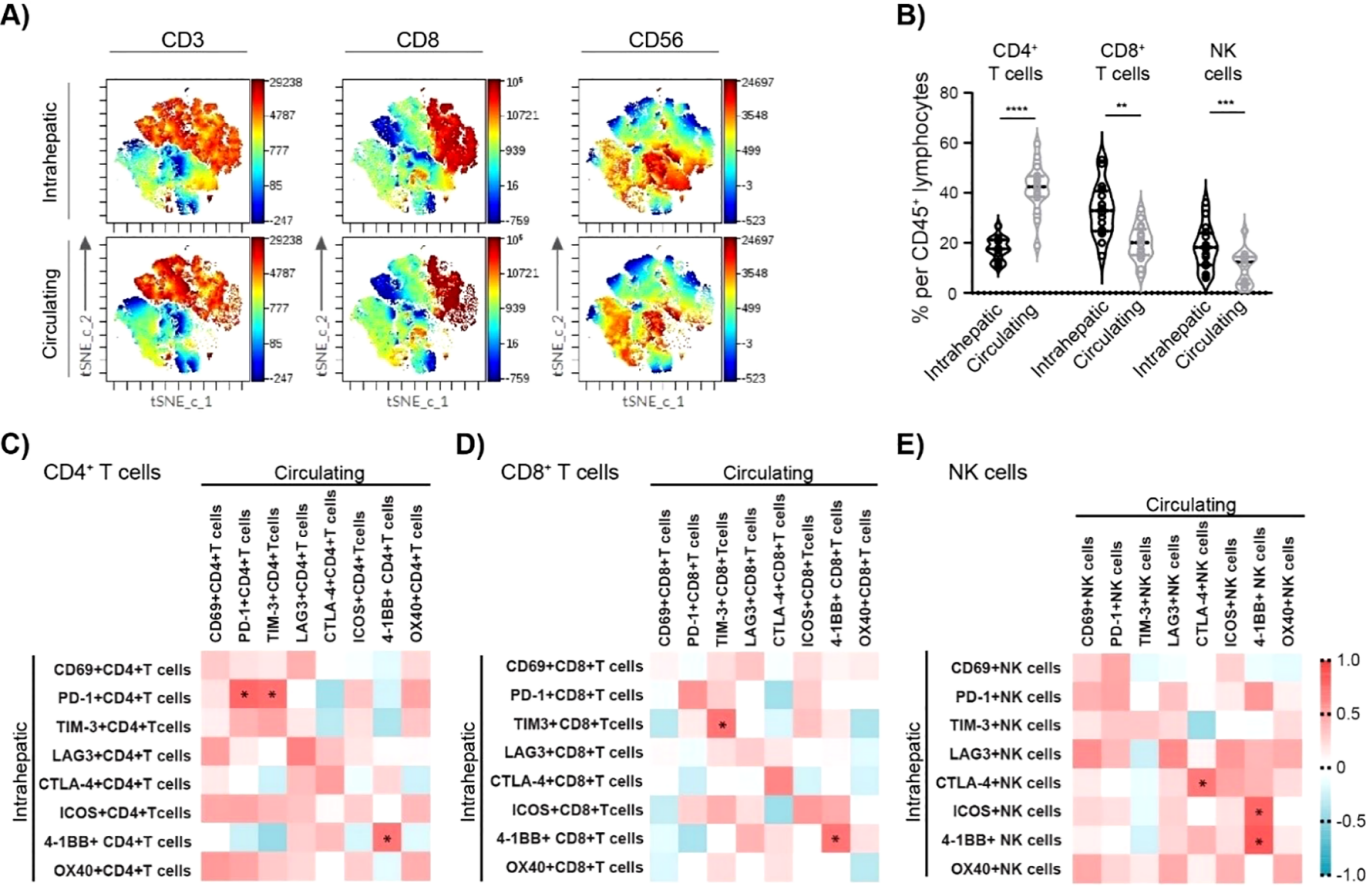
Association between intrahepatic and circulating lymphocyte characteristics of cHBV patients. **(A)** Representative t-SNE-CUDA visualizing intrahepatic and circulating CD45^+^ cells. **(B)** The frequency of CD4^+^ T cells, CD8^+^ T cells, and NK cells per CD45^+^ lymphocytes in the liver and in the blood of cHBV patients (n = 16). Data are visualized as the violin plots showing median and quartile data. Each circle represents a patient. **p < 0.01, ***p < 0.001, ****p < 0.0001 between groups (paired t-test). **(C)** Heatmap of correlation between intrahepatic and circulating CD4^+^ T-cell characteristics. **(D)** Heatmap of correlation between intrahepatic and circulating CD8^+^ T-cell characteristics. **(E)** Heatmap of correlation between intrahepatic and circulating NK cell characteristics. Color corresponds to Spearman’s correlation coefficient r, n = 16. *p < 0.05. cHBV, chronic hepatitis B virus.

To determine the possible correlations between the phenotype of intrahepatic and circulating immune cells in cHBV patients, we investigated paired samples from 16 cHBV patients, for whom both a liver biopsy and a blood sample were collected. As expected, intrahepatic lymphocytes were enriched by CD8^+^ T cells compared to the circulation (**Figures 4A, B**), while CD4^+^ T cells dominated in circulating CD45^+^ lymphocytes of cHBV patients (**Figure 4B**). Similarly to intrahepatic CD8^+^ T cells, NK cells were strongly enriched in the liver compared to the circulation (**Figures 4A, B**).

The association between intrahepatic and circulating ICM expression was measured by Spearman’s rank correlation. A strong positive correlation was observed between the frequency of intrahepatic and circulating PD-1^+^CD4^+^ T cells: r = 0.779, p = 0.001 (**Figure 4C**). Similarly, a positive correlation was observed between the frequency of intrahepatic and circulating TIM-3^+^CD8^+^ T cells (r = 0.718, p = 0.002) (**Figure 4D**), as well as CTLA4^+^ NK cells (r = 0.697, p = 0.004) (**Figure 4E**). Finally, 4-1BB^+^ frequency strongly correlated between intrahepatic and circulating CD4^+^ T cells (r = 0.709, p = 0.003) as well as CD8^+^ T cells (r = 0.721, p = 0.002) and NK cells (r = 0.838,p < 0.001) (**Supplementary Figures S4A–C**). Thus, the circulating profile of 4-1BB expression can provide a relatively precise image of intrahepatic 4-1BB expression.

**Figure 5:**
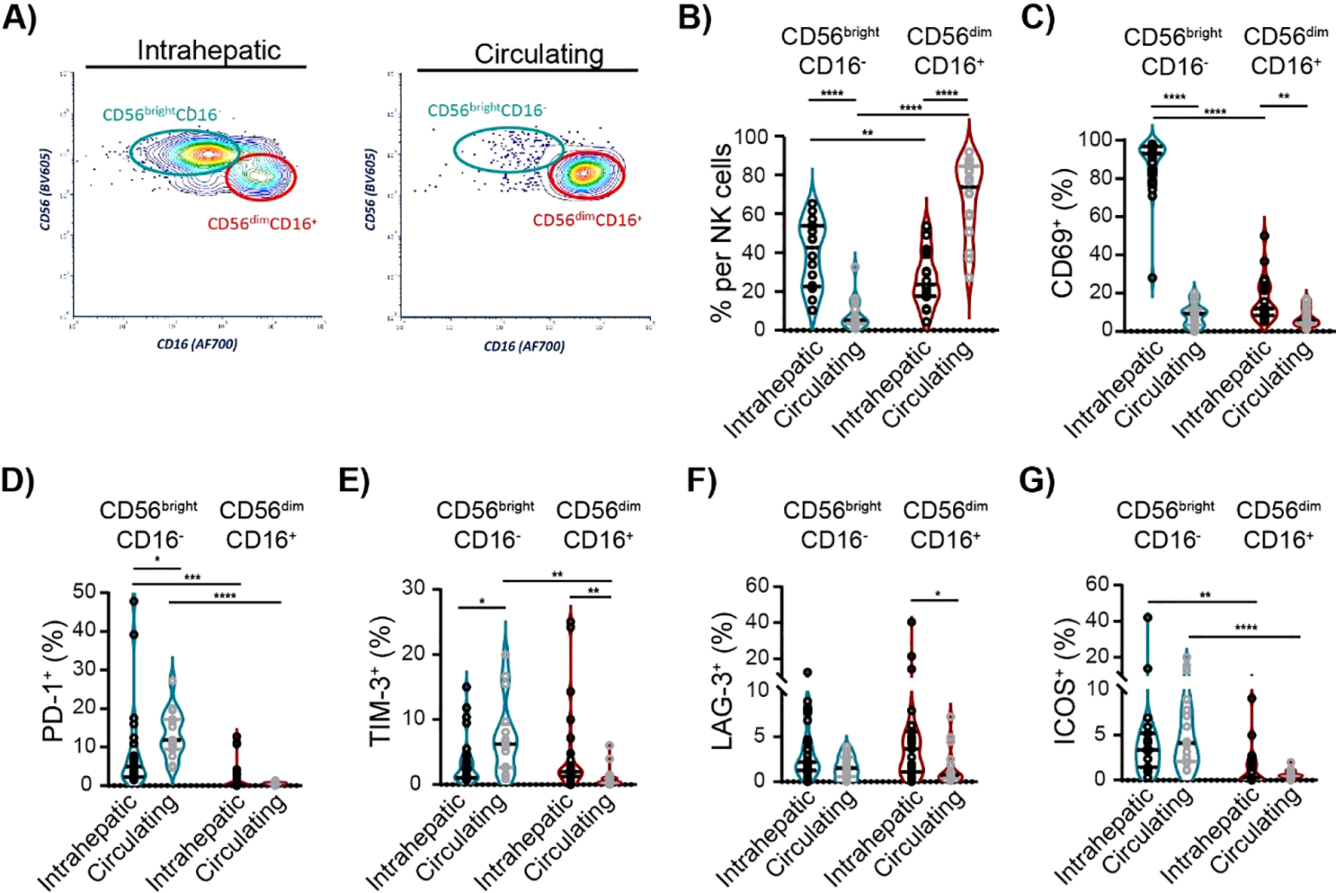
Characteristics of intrahepatic and circulating NK cell subsets of cHBV patients. **(A)** Representative flow cytometry contour plots of intrahepatic and circulating CD56^bright^CD16^−^ and CD56^dim^CD16^+^ NK subsets. **(B)** The frequency of CD56^bright^CD16^−^ and CD56^dim^CD16^+^ cells per NK cells of cHBV patients. Paired, non-parametric Wilcoxon matched-pairs signed-rank test, n = 16. **(C)** The frequency of CD69^+^ cells per NK cell subsets. **(D–G)** The frequency of PD-1^+^ cells, TIM-3^+^ cells, LAG-3^+^ cells, and ICOS^+^ cells per NK cell subsets. Data are visualized as the violin plots showing median and quartile data. Each circle represents a patient. Non-parametric Wilcoxon matched-pairs signed-rank test for paired data; Mann–Whitney test for unpaired data, *p < 0.05, **p < 0.01, ***p < 0.001, ****p < 0.0001 between groups. cHBV, chronic hepatitis B virus.

Next, we investigated the characteristics of intrahepatic and circulating NK cell subsets in cHBV patients. In humans, two main distinct subsets of NK cells are recognized based on their expression of CD56 and CD16: CD56^bright^CD16^−^ and CD56^dim^CD16^+^ NK cells (26). As expected, the majority of intrahepatic NK cells in cHBV patients displayed CD56^bright^CD16^−^ phenotype, while CD56^dim^CD16^+^ NK cells represented the vast majority of circulating NK cells (**Figures 5A, B**; **Supplementary Figures S5A, B**). Importantly, 92.9% of intrahepatic CD56^bright^CD16^−^ NK cells were positive for activation marker CD69 (**Figure 5C**; **Supplementary Figure S5A**). However, only 8.3% of circulating CD56^bright^CD16^−^ NK cells expressed CD69, which was similar to the frequency of CD69^+^ cells in intrahepatic CD56^dim^CD16^+^ NK cell population (9.3%) or in circulating CD56^dim^CD16^+^ NK cell population (6.1%). These results clearly show the significant difference between intrahepatic and circulating NK cell subsets in cHBV patients. The analysis of NK cell subsets also revealed that the main population of intrahepatic NK cells (CD56^bright^CD16^−^) expresses PD-1^+^, while the largest population of circulating NK cells (CD56^dim^CD16^+^ circulating NK cell subset) is PD-1 negative (**Figure 5D**). Similarly, the expression of TIM-3, LAG-3, and ICOS on circulating CD56^dim^CD16^+^ NK cells was negligible compared to that of circulating CD56^bright^CD16^−^ NK and intrahepatic NK cells (**Figures 5E–G**). However, CTLA-4, 4-1BB, and OX40 expression did not differ between NK subsets (**Supplementary Figure S5C**). Thus, although there is a certain correlation between ICM expression on the intrahepatic and circulating lymphocytes, the specific characteristics of intrahepatic NK subsets confirm the need for research directly on liver biopsies from HBV-infected patients.

## 4 Discussion

Both T cells and NK cells are considered to play an irreplaceable role in the elimination of HBV and determine the outcome of HBV infection. ICMs seem to be at the center of the pathogenesis of HBV infection and have a promising potential for clinical translations as targets for therapies. Indeed, the dysregulation of certain ICMs in cHBV patients was previously described on circulating T and NK cells. However, their expression on lymphocytes of the liver, which is the main site of the disease, has been less studied, thus limiting the objective view of their importance in the actual pathology (10).

In this study, we characterized the expression of ICMs (PD-1, TIM-3, LAG-3, CTLA-4, ICOS, 4-1BB, and OX40) in fresh liver biopsies and revealed major alterations of intrahepatic lymphocytes in cHBV patients according to the clinical stage. Our findings bring new insights into the landscape of immune checkpoints on intrahepatic T and NK cells in the context of cHBV infection.

First, we showed that untreated cHBV patients, in both the chronic infection and chronic hepatitis phases, are characterized by an accumulation of CD8^+^ T cells that highly express the activation marker CD69. In accordance with previous observations (15, 16, 27–29), both groups of untreated HBV patients displayed high frequencies of intrahepatic PD-1^+^CD8^+^ T cells. In contrast, TIM-3, ICOS, and 4-1BB-positive CD8^+^ T cells as well as PD-1, TIM-3, and 4-1BB-positive CD4^+^ T cells were enriched mainly in the liver of chronic hepatitis patients. This observation aligns with previous studies demonstrating that TIM-3 expression is elevated on both CD4^+^ and CD8^+^ circulating T cells during the active chronic hepatitis phase, compared to other phases (30, 31). This increased TIM-3 expression is associated with T-cell exhaustion, contributing to impaired antiviral immune responses during active disease. Interestingly, the liver of chronic hepatitis patients contained also the highest frequency of 4-1BB-positive NK cells.

Importantly, we also highlighted that the patients’ HBeAg status is associated with the specific immune phenotype of intrahepatic CD8^+^ T cells and NK cells, with high expression of ICMs, mainly the co-stimulatory molecule 4-1BB. 4-1BB is known to be expressed by T cells and NK cells upon their activation (32). It binds to a single confirmed ligand (4-1BBL) found on the surface of activated antigen-presenting cells (32), thus providing stimulatory signals leading to IL-2 production and T-cell activation, proliferation, and survival and the generation of memory cells (32–34). A previous study revealed that the combination of PD-1 blockade and 4-1BB activation increased responses of human liver T cells against HBV but not HCV (35). Thus, the 4-1BB/4-1BBL pathway may be a crucial protective mechanism engaged in effector cell responses in the HBV context, and both NK cells and T cells should be considered as targeted immune cells in possible 4-1BB activation therapy. Importantly, a recent study by Andreata et al. demonstrated in HBV-transgenic mice that 4-1BB agonism is the most promising strategy to convert dysfunctional effector cells into antiviral effectors for treating cHBV infections. This finding places 4-1BB at the center of current research interest, with our study being crucial, as it describes 4-1BB expression on intrahepatic lymphocytes in patients across different HBV phases.

In general, limited information exists about the expression of ICMs on NK cells in the context of HBV infections. A recent study by Marotel et al. described slightly upregulated PD-1 expression in peripheral NK cells from HBV patients compared to healthy individuals (36). This is consistent with our results showing that the expression of ICMs on the largest population of circulating NK cells, CD56^dim^CD16^+^ circulating NK cell subset, is negligible and that the increased PD-1 expression is observed rather on CD56^bright^CD16^−^ NK cells. Concerning the intrahepatic NK cells, the main population of NK cells—CD56^bright^CD16^−^ NK cells—can express PD-1 and is responsible for the increased frequency of intrahepatic PD-1^+^ NK cells in untreated HBeAg^+^ patients.

The information about the stimulatory molecule ICOS on intrahepatic NK cells in HBV patients was never reported. Previous studies highlighted the important role of ICOS in NK cell development and effector function, but the exact role of ICOS on human NK cells remains undetermined (10). Our study revealed that ICOS is mainly expressed in intrahepatic NK cells of HBeAg^+^ chronic hepatitis patients and that the expression is linked to the CD56^bright^CD16^−^ NK cells, while the CD56^dim^CD16^+^ NK cell subset seems to be ICOS negative. In that regard, the specific features of intrahepatic NK subsets, which cannot be sampled in blood, confirm the importance of research done directly on core liver biopsies or fine-needle aspirates (37).

NUC therapy induces prolonged suppression of viral replication in patients with cHBV, as evidenced by a reduction in serum HBV DNA and HBsAg levels. It has been reported that PD-1 expression on circulating T cells is normalized in patients with NUC therapy compared to untreated cHBV patients, and this reduction is accompanied by an improvement in the ability of T lymphocytes to secrete IL-12 and IFN-γ (38–43). A recent study revealed that the PD-1 expression was reduced in the liver biopsies of patients 96 weeks after the start of the NUC treatment compared to baseline (44). In addition, Nkongolo et al. described via single-cell sequencing of fine-needle aspiration samples from five untreated cHBV patients a specific hepatotoxic signature of CD8^+^ T cells that is not virus specific but associated with liver damage (18). This cell population expresses, among others, PD-1, LAG-3, TIM-3, and 4-1BB, and disappears after NUC treatment. Similarly, our data showed that the number and the distribution of intrahepatic lymphocytes, mainly CD8^+^ T cells, seem to be normalized in NUC-treated HBV patients. Still, even if our results from NUC-treated HBV patients suggest certain trends, they should be interpreted with caution due to the small sample size and the persistence of unnormalized ALT levels and activity scores in some patients despite treatment. In general, we observed that while the frequency of intrahepatic PD-1^+^, ICOS^+^, and 4-1BB^+^ NK cells was increased in untreated HBeAg^+^ patients compared to controls, NUC treatment partially normalized their frequencies. Thus, such modulation of ICMs on intrahepatic lymphocytes during NUC treatment could be associated with HBV suppression. However, we observed no association between the frequency of 4-1BB^+^ lymphocytes and liver injury.

Previous studies demonstrated that the quantity and function of HBV-specific T cells are associated with the outcome of HBV infection. Unfortunately, due to the small size of the liver biopsies, it was not possible to evaluate HBV-specific CD8^+^ T cells. Likewise, the limited sample size of the fresh core liver biopsy prevented us from conducting additional functional studies of immune cells. The scarcity of cHBV patients undergoing core needle liver biopsies further complicates the ability to recruit larger sample sizes for subgroup analyses, including studies focusing on age or sex/gender differences in HBV infection. This limitation is particularly significant, as prior research has shown that age and sex differences have a substantial impact on lymphocyte phenotype and function in HBV (23, 45–47). In our study, NUC-treated patients were older, as age is the key factor in the decision to initiate antiviral therapy.

In summary, analysis of cHBV patient-derived liver tissue revealed the altered expression of ICMs according to the patients’ clinical stage and the link with the HBeAg status of patients. By analyzing a large panel of intrahepatic ICMs expressed by different immune cells, we show that the liver of untreated cHBV patients is characterized by a high expression of immune checkpoint molecules, mainly PD-1, TIM-3, ICOS, and 4-1BB, providing an attractive approach to investigate their potential role as targets for effective therapeutic interventions.

## Data Availability

The original contributions presented in the study are included in the article/**Supplementary Material**. Further inquiries can be directed to the corresponding author.
