# Supplementary material for: Differences in the intrahepatic expression of immune checkpoint molecules on T cells and natural killer cells in chronic HBV patients
Source: Front Immunol. 2025 Jan 15;15:1489770. doi: 10.3389/fimmu.2024.1489770 (PMC11774737; doi:10.3389/fimmu.2024.1489770)
Supplement: Supplementary file 1 [file DataSheet1.docx]

Supplementary Material

# Materials and Methods

Flow cytometry analyses

Fresh intrahepatic cell suspension and blood samples were stained, without any stimulation, with the following anti-human antibodies:

- **Tube 1** included anti-CD45-APC/Cy7 (clone HI30, BioLegend), anti-CD3-PerCP-Cy5.5 (clone UCHT1, BioLegend), anti-CD56-BV605 (clone HCD56, BioLegend), anti-CD16-AF700 (clone 3G8, BioLegend), anti-CD15-BV510 (clone W6D3, BioLegend), anti-CD19-APC (clone HIB19, BioLegend), anti-CD8-PE/Cy7 (clone RPA-T8, BD Biosciences), anti-CD69-PE (clone FN50, BioLegend), anti-4-1BB-BV421 (clone 4B4-1, BioLegend), anti ICOS-BV785 (cloneC398.4A) and anti-PD-1-BV711 (clone EH12.2H7, BioLegend).
- **Tube 2** included anti-CD45-APC/Cy7 (clone HI30, BioLegend), anti-CD3-PerCP-Cy5.5 (clone UCHT1, BioLegend), anti-CD56-BV605 (clone HCD56, BioLegend), anti-CD16-AF700 (clone 3G8, BioLegend), anti-CD15-BV510 (clone W6D3, BioLegend), anti-CD19-APC (clone HIB19, BioLegend), anti-CD8-PE/Cy7 (clone RPA-T8, BD Biosciences), anti-LAG3-PE (clone 3DS223H, eBioscience), anti-OX40-FITC (clone Ber-ACT35, BioLegend), anti-CTLA4-BV421 (clone BNI3, BioLegend), anti-TIM3-BV785 (clone F39-2E2, BioLegend).
- The fluorescence minus one (FMO) controls were used to determine positive populations. Fixable Viability kit (PE-Texas Red-A channel) was used to exclude dead cells and FluoroFix™ Buffer (Biolegend) was used for fixation to stabilize tandem dyes. Data were acquired on BD-LSRII flow cytometer (BD Biosciences, Le Pont-De-Claix, France), collected with BD FACSDiva 6.3.1 software and analyzed using FCS Express 7 Flow software and Cytobank. The Spillover spreading for fluorophores used in panels is summarized in Supplementary Table 4. The gating strategy (Figure S1) allowed to separate main populations of lymphocytes. CD45^+^CD56^-^CD3^+^ cells were considered as T cells, and within this population, CD8^-^ cells were considered as CD4^+^ T cells.

# Supplementary Figures and Tables

## Supplementary Figures

| **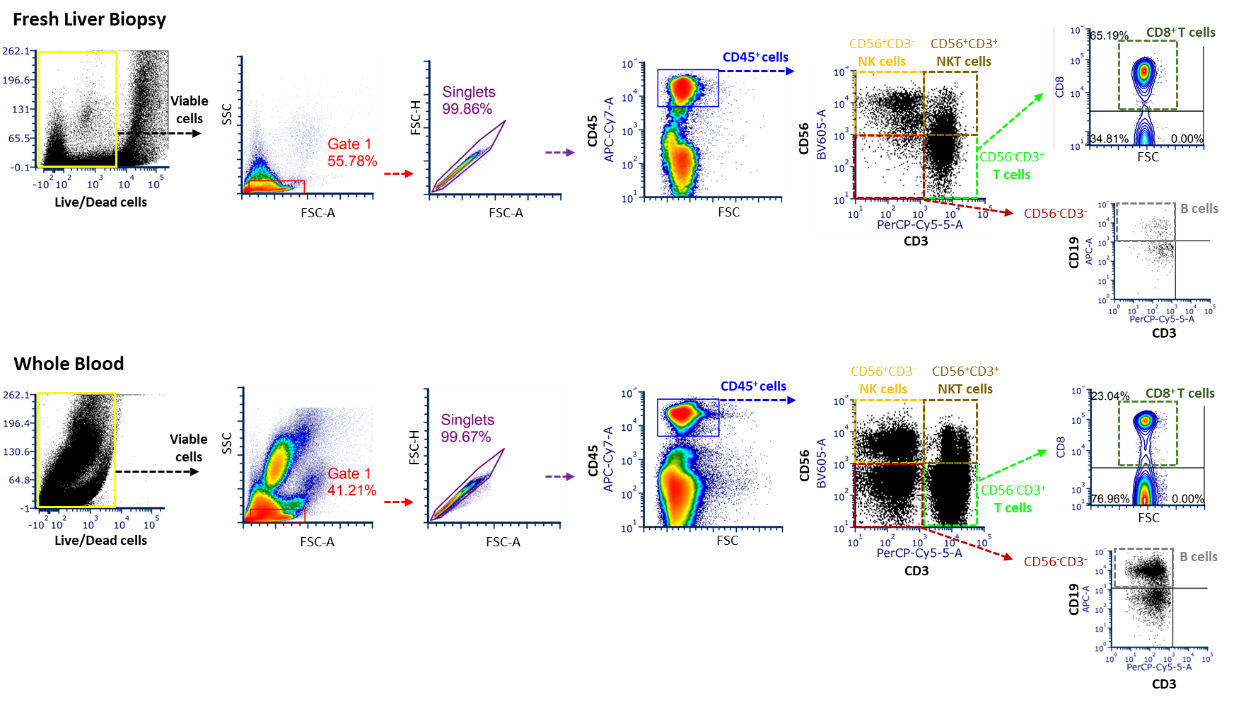** |
| --- |
| **Supplementary Figure 1.** **Gating strategy used throughout the experiments for flow cytometry analysis.**  Fresh samples of liver biopsies and whole blood were stained for FACS analyses. After exclusion of dead cells, lymphocytes were gated based on SSC and FSC and doublets were excluded. Then, based on CD45 expression, we identified CD45+ lymphocytes that were further gated to CD3-CD56+ NK cells, CD3+CD56+ NKT cells and CD3+CD56- classical T cells that were further gated on CD8+ T cells. CD3-CD56- cells were gated based on CD19 as B cells. |
|  |
|  |
|  |
|  |
|  |
|  |
|  |
|  |
| 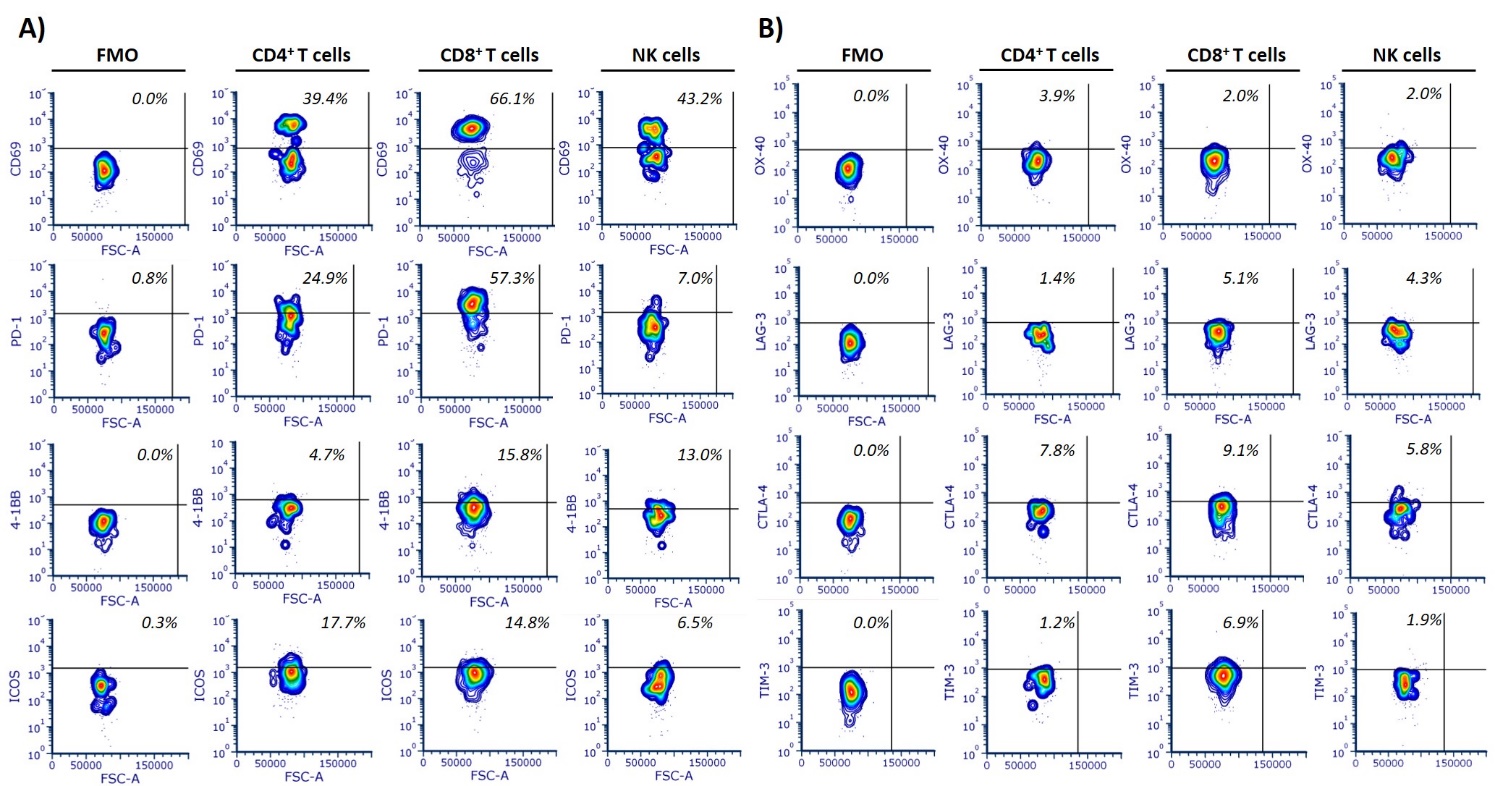 |
| **Supplementary Figure 2.** **Representative flow cytometry plots of CD69 positive and immune checkpoint molecules positive cells, including FMO controls.** A) Representative flow cytometry plots of markers stained in tube 1 for CD4+ and CD8+ T cells, and NK cells. B) Representative flow cytometry plots of markers stained in tube 2 for CD4+ and CD8+ T cells and NK cells.   \| 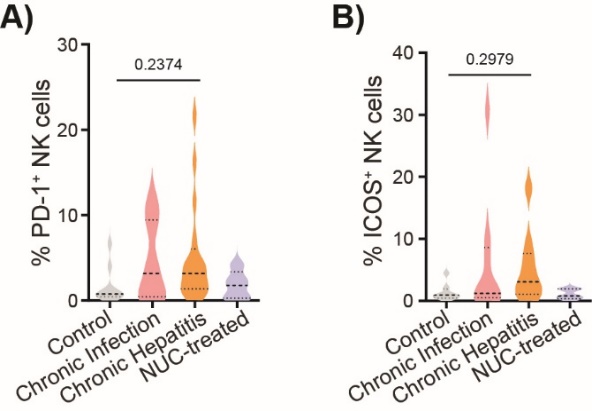 \| \| --- \| \| **Supplementary Figure 3. Expression of ICM on intrahepatic NK cells in HBV patients.** (A) The frequency of PD-1^+^ NK cells. (B) The frequency of ICOS^+^ NK cells. Data are visualized as the violin plots showing median and quartile data. Control (n = 10), cHBV untreated Chronic Infection (n = 9) and Chronic Hepatitis (n = 16), and NUC-treated HBV (n = 5). Kruskal-Wallis test with Dunn multiple comparison post-test. \| |

| 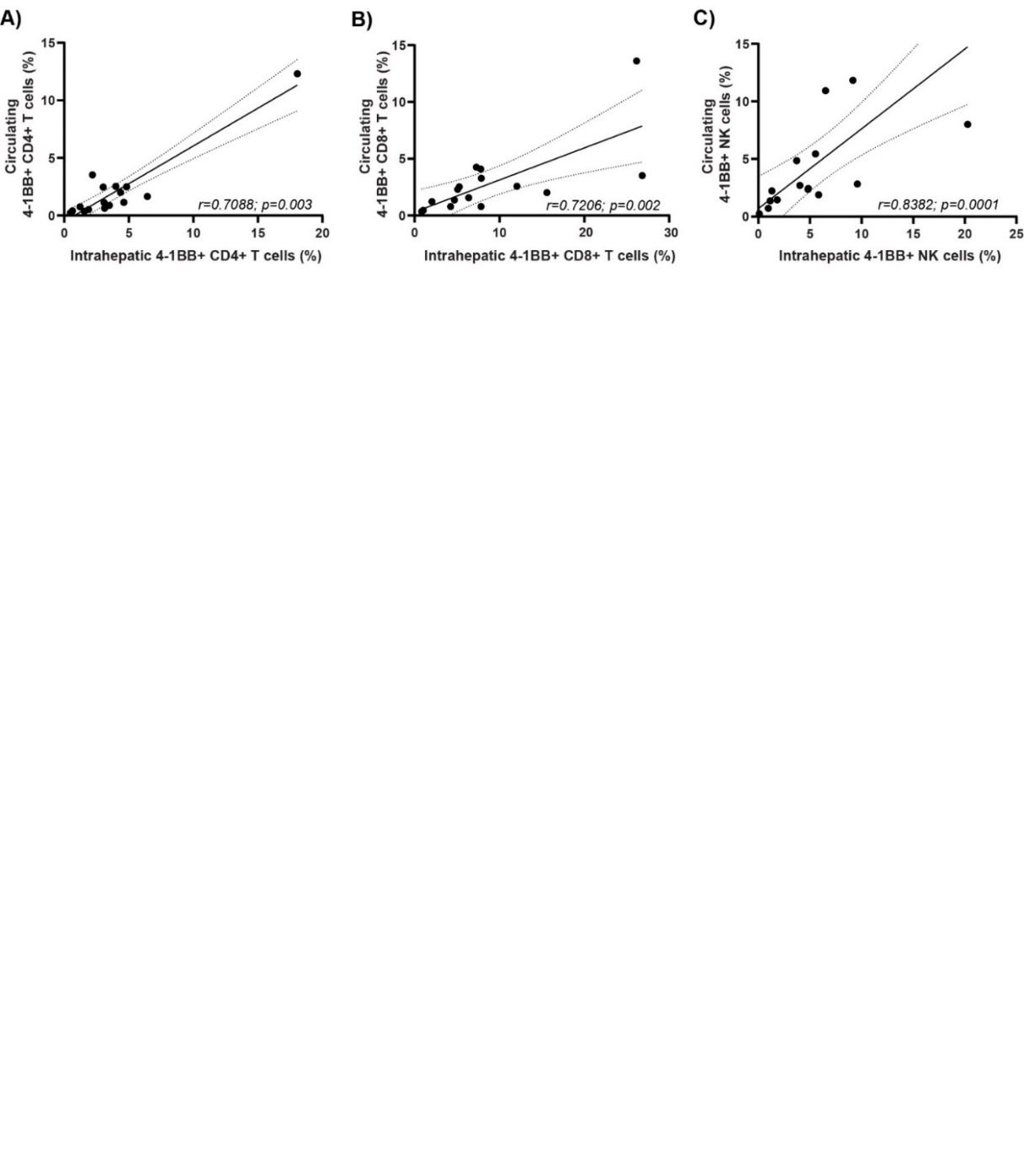 |
| --- |
| **Supplementary Figure 4.** **Association between intrahepatic and circulating characteristics in untreated cHBV patients.** A) Correlation between circulating and intrahepatic 4-1BB+CD4+ T cells. (B) Correlation between circulating and intrahepatic 4-1BB+ CD8+ T cells. (C) Correlation between circulating and intrahepatic 4-1BB+ NK cells. Each circle represents a patient. Spearman correlation coefficient r, n = 16. |
|  |
|  |
| 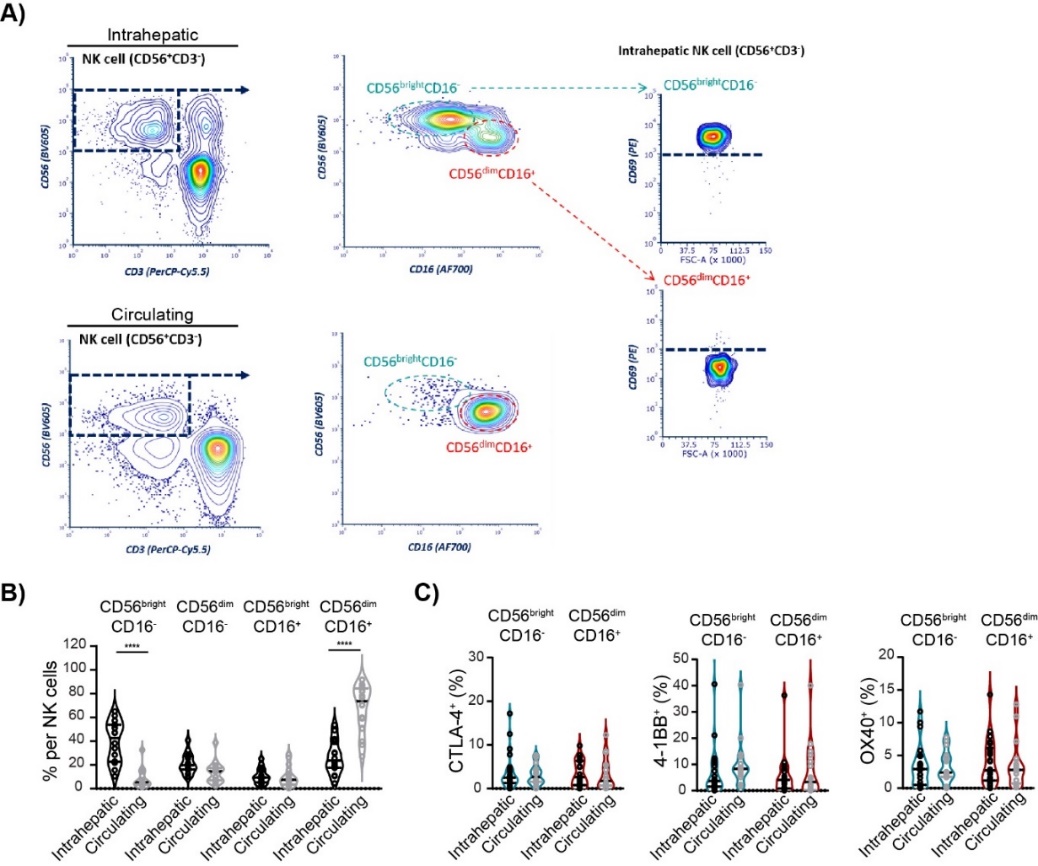  **Supplementary Figure 5.** **Characteristics of intrahepatic and circulating NK cell subsets in HBV-infected patients.** A) Representative contour plot of flow cytometry gating strategy for CD56brightCD16- and CD56dimCD16+ NK cells. (B) The frequency of CD56brightCD16-, CD56dimCD16-, CD56brightCD16+ and CD56dimCD16+ cells per NK cells of cHBV patients. Paired, nonparametric Wilcoxon matched-pairs signed rank test, n=16). (C) The frequencies of CTLA-4+, 4-1BB+ and OX40+ cells per NK cell subsets. Data are visualized as the violin plots showing median and quartile data. Each circle represents a patient. Nonparametric Wilcoxon matched-pairs signed rank test for paired data, **** p < 0.0001 between groups. |

## Supplementary Tables

**Supplementary table S1. Clinical, biological and histological features of HBV patients with paired fresh liver biopsy and blood sample**

|  | n = 16 |
| --- | --- |
| Chronic Infection/ Chronic Hepatitis/ NUC-treated | 2/11/3 |
| Sex (M/F) | 9/7 |
| Age, median [IQR] | 34.5 [27.3-48.3] |
| ALT (U/L), median [IQR] | 46.0 [26.5-54.5] |
| AST (U/L), median [IQR] | 38.5 [27.5-52.0] |
| HBeAg, n (%)  Negative  Positive | 13 (81.3%)  3 (18.7%) |
| HBsAg (UI/mL), median [IQR] | 8664 [396-17716] |
| HBV DNA (log_10_ UI/mL), median [IQR] | 3.51 [3.05-5.03] |
| Antiviral treatment | 3 (18.7%) |
| Stage of fibrosis  F0/F1/F2/F3-F4 | 2/10/3/1 |
| Activity score  A0/A1/A2/A3 | 2/11/2/1 |

Alanine transaminase, ALT; Aspartate transaminase, AST; hepatitis Be antigen, HBeAg; hepatitis B surface antigen, HBsAg, NA; not applicable. Values are presented as median [IQR, 25th-75th percentile].

**Supplementary table S2. The number and frequency of intrahepatic immune cells**

|  | **Controls**  n = 10 | **Chronic Infection**  (former Inactive Carriers)  n = 9 | **Chronic Hepatitis**  n = 16 | **NUC-treated**  n = 5 | *Kruskal-Wallis test, p* value |
| --- | --- | --- | --- | --- | --- |
|  |  |  |  |  |  |
| CD4^+^ T cells per mg, *median [IQR]* | 208  [92-578] | 237  [163-422] | 233  [133-608] | 294  [149-638] | 0.9420 |
| CD8^+^ T cells per mg, *median [IQR]* | 264  [184-715] | 525  [354-865] | 620  [269-1417] | 232  [132-588] | 0.2199 |
| NKT cells per mg, *median [IQR]* | 290  [177-371] | 258  [223-805] | 301  [101-416] | 293  [106-758] | 0.9039 |
| NK cells per mg, *median [IQR]* | 327  [217-485] | 319  [121-607] | 251  [113-536] | 294  [192-458] | 0.9542 |
| B cells per mg, *median [IQR]* | 50.2  [33.9-68.8] | 28.4  [15.6-46.3] | 24.3  [13.5-53.6] | 20.9  [12.8-47.2] | 0.3274 |
|  |  |  |  |  |  |
| % of CD45^+^ cells, *median [IQR]* |  |  |  |  |  |
| T cells | 44.4  [34.9-51.6] | 48.04  [45.7-58.2] | 53.5  [45.5-65.4] | 44.7  [33.9-52.2] | 0.1812 |
| CD4^+^ T cells | 17.5  [13.8-22.2] | 17.3  [13.0-17.7] | 17.9  [15.1-21.2] | 20.7  [17.7-26.9] | 0.1880 |
| CD8^+^ T cells | 24.3  [18.7-33.7] | 31.7  [29.7-40.8] | 32.1  [26.3-45.3] | 19.6  [16.3-27.5] | 0.0136 |
| NKT cells | 22.7  [14.6-28.6] | 21.1  [16.6-26.1] | 15.4  [9.7-28.7] | 20.2  [13.2-30.2] | 0.6071 |
| NK cells | 21.3  [13.3-30.1] | 18.8  [16.3-22.1] | 18.7  [11.9-23.1] | 25.0  [15.0-38.4] | 0.5274 |
| B cells | 3.75  [2.33-4.20] | 1.50  [1.25-2.20] | 1.90  [1.20-2.95] | 1.70  [1.30-3.30] | 0.0655 |
| % of T cells, *median [IQR]* |  |  |  |  |  |
| CD4^+^ T cells | 36.8  [31.6-47.6] | 30.9  [28.3-36.3] | 34.2  [26.9-41.1] | 53.5  [44.0-57.9] | 0.0061 |
| CD8^+^ T cells | 63.3  [52.4-68.4] | 69.1  [63.5-72.1] | 65.8  [59.0-73.1] | 46.5  [42.2-56.1] | 0.0061 |

Values are presented as median [IQR, 25th-75th percentile].

**Supplementary table S3. Clinical, biological and histological features of untreated Immune Active cHBV patients**

| Parameters | **Chronic Hepatitis HBeAg-**  n = 11 | **Chronic Hepatitis HBeAg+**  n = 5 |
| --- | --- | --- |
| Sex (M/F) | 9/2 | 5/0 |
| Age, median [IQR] | 29.0 [19.0-38.0] | 27.0 [20.0-29.5] |
| ALT (U/L), median [IQR] | 53.0 [34.0-64.0] | 47.0 [42.5-211.0] |
| AST (U/L), median [IQR] | 40.0 [31.0-45.0] | 49.0 [31.0-157.0] |
| HBeAg, n (%)  Negative  Positive | 11 (100%)  0 (0%) | 0 (0%)  5 (100%) |
| HBsAg (UI/mL), median [IQR] | 14746 [4472-17716] | 17031 [2958-122500] |
| HBV DNA (log_10_ UI/mL), median [IQR] | 3.72 [3.27-4.38] | 7.81 [5.17-8.52] |
| Antiviral treatment  Entecavir  Tenofovir | 0  0 | 0  0 |
| Stage of fibrosis  F0/F1/F2/F3/F4 | 2/6/2/1/0 | 1/2/2/0/0 |
| Activity score  A0/A1/A2/A3 | 0/11/0/0 | 0/3/1/1 |

Alanine transaminase, ALT; Aspartate transaminase, AST; hepatitis Be antigen, HBeAg; hepatitis B surface antigen, HBsAg, NA; not applicable. Values are presented as median [IQR, 25th-75th percentile].

**Supplementary table S4. Spillover data**

| **Target - % Source** | **Value** |
| --- | --- |
| FITC-A - % FITC-A | 1.000 |
| PE-A - % FITC-A | -0.206 |
| PerCP-Cy5-5-A - % FITC-A | 0.005 |
| PE-Cy7-A - % FITC-A | -0.001 |
| APC-A - % FITC-A | 0.000 |
| Alexa Fluor 700-A - % FITC-A | 0.000 |
| APC-Cy7-A - % FITC-A | 0.000 |
| BV421-A - % FITC-A | 0.005 |
| BV510-A - % FITC-A | -0.048 |
| BV605-A - % FITC-A | 0.034 |
| BV711-A - % FITC-A | -0.002 |
| BV786-A - % FITC-A | 0.000 |
| PE-Texas Red-A - % FITC-A | 0.000 |
| FITC-A - % PE-A | -0.008 |
| PE-A - % PE-A | 1.002 |
| PerCP-Cy5-5-A - % PE-A | -0.117 |
| PE-Cy7-A - % PE-A | 0.010 |
| APC-A - % PE-A | 0.002 |
| Alexa Fluor 700-A - % PE-A | 0.002 |
| APC-Cy7-A - % PE-A | 0.000 |
| BV421-A - % PE-A | 0.000 |
| BV510-A - % PE-A | -0.002 |
| BV605-A - % PE-A | -0.080 |
| BV711-A - % PE-A | 0.029 |
| BV786-A - % PE-A | -0.002 |
| PE-Texas Red-A - % PE-A | 0.000 |
| FITC-A - % PerCP-Cy5-5-A | 0.000 |
| PE-A - % PerCP-Cy5-5-A | 0.002 |
| PerCP-Cy5-5-A - % PerCP-Cy5-5-A | 1.031 |
| PE-Cy7-A - % PerCP-Cy5-5-A | -0.192 |
| APC-A - % PerCP-Cy5-5-A | -0.019 |
| Alexa Fluor 700-A - % PerCP-Cy5-5-A | -0.023 |
| APC-Cy7-A - % PerCP-Cy5-5-A | 0.003 |
| BV421-A - % PerCP-Cy5-5-A | 0.008 |
| BV510-A - % PerCP-Cy5-5-A | 0.000 |
| BV605-A - % PerCP-Cy5-5-A | 0.000 |
| BV711-A - % PerCP-Cy5-5-A | -0.221 |
| BV786-A - % PerCP-Cy5-5-A | 0.021 |
| PE-Texas Red-A - % PerCP-Cy5-5-A | 0.000 |
| FITC-A - % PE-Cy7-A | -0.001 |
| PE-A - % PE-Cy7-A | -0.011 |
| PerCP-Cy5-5-A - % PE-Cy7-A | -0.002 |
| PE-Cy7-A - % PE-Cy7-A | 1.005 |
| APC-A - % PE-Cy7-A | 0.005 |
| Alexa Fluor 700-A - % PE-Cy7-A | 0.000 |
| APC-Cy7-A - % PE-Cy7-A | -0.047 |
| BV421-A - % PE-Cy7-A | 0.003 |
| BV510-A - % PE-Cy7-A | 0.000 |
| BV605-A - % PE-Cy7-A | 0.000 |
| BV711-A - % PE-Cy7-A | 0.001 |
| BV786-A - % PE-Cy7-A | -0.037 |
| PE-Texas Red-A - % PE-Cy7-A | 0.000 |
| FITC-A - % APC-A | 0.000 |
| PE-A - % APC-A | 0.000 |
| PerCP-Cy5-5-A - % APC-A | -0.001 |
| PE-Cy7-A - % APC-A | 0.001 |
| Alexa Fluor 700-A - % APC-A | -0.113 |
| APC-Cy7-A - % APC-A | 0.001 |
| BV421-A - % APC-A | 0.000 |
| BV510-A - % APC-A | 0.000 |
| BV605-A - % APC-A | 0.000 |
| BV711-A - % APC-A | 0.000 |
| BV786-A - % APC-A | 0.001 |
| PE-Texas Red-A - % APC-A | 0.000 |
| FITC-A - % Alexa Fluor 700-A | -0.002 |
| PE-A - % Alexa Fluor 700-A | 0.000 |
| PerCP-Cy5-5-A - % Alexa Fluor 700-A | -0.029 |
| PE-Cy7-A - % Alexa Fluor 700-A | 0.006 |
| APC-A - % Alexa Fluor 700-A | 0.023 |
| Alexa Fluor 700-A - % Alexa Fluor 700-A | 1.065 |
| APC-Cy7-A - % Alexa Fluor 700-A | -0.255 |
| BV421-A - % Alexa Fluor 700-A | 0.003 |
| BV510-A - % Alexa Fluor 700-A | 0.001 |
| BV605-A - % Alexa Fluor 700-A | 0.000 |
| BV711-A - % Alexa Fluor 700-A | -0.123 |
| BV786-A - % Alexa Fluor 700-A | 0.022 |
| PE-Texas Red-A - % Alexa Fluor 700-A | 0.000 |
| FITC-A - % APC-Cy7-A | 0.000 |
| PE-A - % APC-Cy7-A | 0.000 |
| PerCP-Cy5-5-A - % APC-Cy7-A | 0.004 |
| PE-Cy7-A - % APC-Cy7-A | -0.031 |
| APC-A - % APC-Cy7-A | -0.105 |
| Alexa Fluor 700-A - % APC-Cy7-A | -0.150 |
| APC-Cy7-A - % APC-Cy7-A | 1.054 |
| BV421-A - % APC-Cy7-A | 0.005 |
| BV510-A - % APC-Cy7-A | -0.001 |
| BV605-A - % APC-Cy7-A | 0.001 |
| BV711-A - % APC-Cy7-A | 0.019 |
| BV786-A - % APC-Cy7-A | -0.071 |
| PE-Texas Red-A - % APC-Cy7-A | 0.000 |
| FITC-A - % BV421-A | 0.000 |
| PE-A - % BV421-A | 0.000 |
| PerCP-Cy5-5-A - % BV421-A | 0.000 |
| PE-Cy7-A - % BV421-A | 0.000 |
| APC-A - % BV421-A | 0.000 |
| Alexa Fluor 700-A - % BV421-A | 0.000 |
| APC-Cy7-A - % BV421-A | 0.000 |
| BV421-A - % BV421-A | 1.010 |
| BV510-A - % BV421-A | -0.082 |
| BV605-A - % BV421-A | 0.034 |
| BV711-A - % BV421-A | 0.000 |
| BV786-A - % BV421-A | 0.000 |
| PE-Texas Red-A - % BV421-A | 0.000 |
| FITC-A - % BV510-A | -0.003 |
| PE-A - % BV510-A | 0.001 |
| PerCP-Cy5-5-A - % BV510-A | 0.006 |
| PE-Cy7-A - % BV510-A | 0.000 |
| APC-A - % BV510-A | 0.001 |
| Alexa Fluor 700-A - % BV510-A | -0.001 |
| APC-Cy7-A - % BV510-A | 0.000 |
| BV421-A - % BV510-A | -0.123 |
| BV510-A - % BV510-A | 1.011 |
| BV605-A - % BV510-A | -0.295 |
| BV711-A - % BV510-A | 0.008 |
| BV786-A - % BV510-A | -0.001 |
| PE-Texas Red-A - % BV510-A | 0.000 |
| FITC-A - % BV605-A | 0.000 |
| PE-A - % BV605-A | -0.004 |
| PerCP-Cy5-5-A - % BV605-A | 0.000 |
| PE-Cy7-A - % BV605-A | 0.002 |
| APC-A - % BV605-A | -0.003 |
| Alexa Fluor 700-A - % BV605-A | 0.015 |
| APC-Cy7-A - % BV605-A | 0.001 |
| BV421-A - % BV605-A | -0.011 |
| BV510-A - % BV605-A | -0.001 |
| BV605-A - % BV605-A | 1.001 |
| BV711-A - % BV605-A | -0.113 |
| BV786-A - % BV605-A | 0.009 |
| PE-Texas Red-A - % BV605-A | 0.000 |
| FITC-A - % BV711-A | 0.000 |
| PE-A - % BV711-A | 0.000 |
| PerCP-Cy5-5-A - % BV711-A | -0.134 |
| PE-Cy7-A - % BV711-A | -0.001 |
| APC-A - % BV711-A | 0.000 |
| Alexa Fluor 700-A - % BV711-A | -0.132 |
| APC-Cy7-A - % BV711-A | 0.000 |
| BV421-A - % BV711-A | -0.021 |
| BV510-A - % BV711-A | 0.000 |
| BV605-A - % BV711-A | 0.001 |
| BV711-A - % BV711-A | 1.055 |
| BV786-A - % BV711-A | -0.205 |
| PE-Texas Red-A - % BV711-A | 0.000 |
| FITC-A - % BV786-A | 0.000 |
| PE-A - % BV786-A | 0.001 |
| PerCP-Cy5-5-A - % BV786-A | 0.004 |
| PE-Cy7-A - % BV786-A | -0.086 |
| APC-A - % BV786-A | 0.004 |
| Alexa Fluor 700-A - % BV786-A | 0.003 |
| APC-Cy7-A - % BV786-A | -0.052 |
| BV421-A - % BV786-A | -0.084 |
| BV510-A - % BV786-A | -0.006 |
| BV605-A - % BV786-A | -0.003 |
| BV711-A - % BV786-A | -0.038 |
| BV786-A - % BV786-A | 1.017 |
| PE-Texas Red-A - % BV786-A | 0.000 |
| FITC-A - % PE-Texas Red-A | 0.000 |
| PE-A - % PE-Texas Red-A | 0.000 |
| PerCP-Cy5-5-A - % PE-Texas Red-A | 0.000 |
| PE-Cy7-A - % PE-Texas Red-A | 0.000 |
| APC-A - % PE-Texas Red-A | 0.000 |
| Alexa Fluor 700-A - % PE-Texas Red-A | 0.000 |
| APC-Cy7-A - % PE-Texas Red-A | 0.000 |
| BV421-A - % PE-Texas Red-A | 0.000 |
| BV510-A - % PE-Texas Red-A | 0.000 |
| BV605-A - % PE-Texas Red-A | 0.000 |
| BV711-A - % PE-Texas Red-A | 0.000 |
| PE-Texas Red-A - % PE-Texas Red-A | 1.000 |
